# Early changes in peripheral blood cytokine levels after the treatment of metastatic hepatic carcinoma with CalliSpheres microspheres drug-eluting beads transcatheter arterial chemoembolization

**DOI:** 10.3389/fonc.2022.889312

**Published:** 2022-07-29

**Authors:** Ying Liu, Song Liu, Guang Sheng Zhao, Xiang Li, Fei Gao, Zhi Zhong Ren, Jie Bian, Jian Lin Wu, Yue Wei Zhang

**Affiliations:** ^1^ Hepatopancreatobiliary Center, Beijing Tsinghua Changgung Hospital, Beijing, China; ^2^ Interventional Medicine Center, Linyi Cancer Hospital, Linyi, China; ^3^ Minimally Invasive Interventional Treatment Center, Affiliated Zhongshan Hospital of Dalian University, Dalian, China; ^4^ Cancer Treatment Center, Affiliated Zhongshan Hospital of Dalian University, Dalian, China; ^5^ Department of Interventional Oncology, The Second Hospital of Dalian Medical University, Dalian, China; ^6^ Department of Radiology, The Second Hospital of Dalian Medical University, Dalian, China; ^7^ Department of Radiology, Affiliated Zhongshan Hospital of Dalian University, Dalian, China

**Keywords:** CalliSpheres microspheres, CSM-TACE, liver metastatic carcinoma, Th1/Th2, IL-17A

## Abstract

**Objective:**

To observe the early changes in peripheral blood cytokine levels after treatment of metastatic hepatic carcinoma (MHC) with CalliSpheres microspheres drug-eluting beads (DEB) transcatheter arterial chemoembolization (CSM-TACE).

**Methods:**

Twenty-eight patients with refractory MHC who underwent CSM-TACE were selected prospectively, and 5mL of peripheral blood was collected before CSM-TACE and on the 2nd and 5th day after CSM-TACE. Flow cytometry was used to detect immunological indicators. The early changes in levels of peripheral blood cell inflammatory factors Th1 (interleukin 2 (IL-2), tumor necrosis factor-α (TNF-a), interferon (IFN-r)), Th2 (IL-4, IL-6, IL-10), and Th17 (IL-17A) were observed after CSM-TACE, as well as the ratio of CD4^+^/CD8^+^.

**Results:**

All the 28 patients underwent CSM-TACE successfully. CT at 4 days after CSM-TACE showed clear outline low-density changes in liver tumors, and honeycomb necrosis was observed in the tumors in some cases. After CSM-TACE, the IL-6 and IL-10 levels were increased and then decreased again. After CSM-TACE, IL-2 showed a trend of transient increase and then decreased again, and the TNF-a level decreased temporarily, and then decreased. After CSM-TACE, the IFN-r level showed a continuous and slowly increasing trend. The IL-17 level showed a continuous downward trend, and the CD4^+^/CD8^+^ ratio showed a gradual and continuous upward trend, and there was a negative correlation between them.

**Conclusions:**

There are complex dynamic changes in TH1/Th2 in the early stage of CSM-TACE, and the acute inflammatory response and the enhancement of the body’s immune anti-tumor response coexist.

## Introduction

For patients with unresectable metastatic hepatic carcinoma (MHC), especially the refractory liver metastatic carcinoma that has progressed after chemotherapy, often die of irreversible liver failure within 3-5 months (1). CalliSpheres microspheres transcatheter arterial chemoembolization (CSM-TACE) with domestic drug-loaded microspheres has achieved good clinical effects in the treatment of various refractory liver cancers, and achieved the same therapeutic effect as imported drug-loaded microspheres (2, 3), and CSM-TACE seems to have more advantages in the treatment of refractory liver metastatic carcinoma (4). TACE for the treatment of primary hepatocellular carcinoma (PHC) can cause a series of changes in the body’s cytokines, thus further changing the tumor microenvironment (5–9). There is no research on the effect of CSM-TACE on the immune-inflammatory factors after the treatment of liver metastatic carcinoma.

TACE with microspheres embolic agent (m-TACE) can caused diffuse and significant necrosis of liver tumors shortly soon (10), and the level of Treg cells decreased after m-TACE, suggesting that m-TACE has a positive immune regulation ability for the treatment of PHC (11). TACE combined with various ablation therapies or immunotherapy for the treatment of PHC can also increase the body’s anti-tumor ability, mainly manifested as CD4^+^, CD4^+^/CD8^+^, NK cell levels increasing, CD8^+^ and Treg cell level decreasing, etc. (12). At present, most scholars mainly focus on the balance of various inflammatory factors and Th1/Th2 as the prognostic indicators of liver cancer after TACE (5, 6, 8), especially IL-6, it is considered to be an important survival predictive factor. Circulating blood IL-17 concentration is also considered to be a predictive factor of hepatocellular carcinoma (HCC) in patients with liver cirrhosis. The combination of alpha-fetoprotein (AFP) and IL-17 has a high sensitivity for predicting HCC within 1 year (13). In the NASH-HCC model, the inflammatory factor FGF21 can inhibit liver cells-TLR4-IL-17-A signaling pathway to prevent the conversion of NASH to HCC (14). miR-132 actively regulates Th17 cell differentiation and improves the tumor promotion effect of Th17 on hematopoietic stem cells (HSCs) (15). In HCC patients with complete or partial tumor response to TACE, the levels of circulating Th1, Th17 and CD4^+^/IFN-γ^+^/IL-17^+^T cells increased (16), suggesting that Th17 is closely related to liver cancer, and may have the “dual identity” of tumor-promoting and anti-tumor effects at the same time (17).

This study prospectively observed the changes in the levels of Th1/Th2 and Th17 and other inflammatory factors in the peripheral blood within 1 week after CSM-TACE treatment of refractory MHC with domestic CalliSpheres drug-loaded microspheres and further explored the effect of CSM-TACE treatment on the recent immune inflammation in MHC patients.

## Materials and methods

### Inclusion criteria

Patients who meet any of the following criteria are considered refractory MHC: 1. Recurrence or progression after surgical resection or ablation; 2. Patients with the lesion that adjacent to the gallbladder and hepatic portal area and cannot or is not suitable for surgery and ablation; 3. Patients with more than three liver internal metastatic lesions or with diffuse liver cancer; 4. Intolerance of systemic chemotherapy or progression after second-line chemotherapy, or progression after immune system therapy.

### Patients’ information

A total of 28 patients with refractory MHC in the minimally invasive treatment center of our hospital underwent CSM-TACE interventional therapy from January 2020 to December 2020. There were 16 males and 12 females, aged from 34 to 77 years, with an average age of 59.9 ± 9.26 years. Sources of primary tumors: 11 cases of colorectal cancer, 5 cases of gastric cancer, 4 cases of pancreatic cancer, 2 cases of breast cancer, 2 cases of ovarian cancer, 2 cases of endometrial cancer, and 2 cases of lung cancer. The number of tumors: 4 cases (1 to 3 tumors), 15 cases (3 to 5 tumors), 9 cases (>5 tumors), and the intrahepatic lesions in patients were evaluated for postoperative tumor response according to mRECIST1.0 standard, and when the number of lesions in the patient was ≥5, 5 lesions were selected as target lesions in order from largest to smallest. The study was approved by the hospital ethics committee. All patients signed an informed consent form before treatment and agreed to CSM-TACE treatment and peripheral blood testing before and after CSM-TACE (**Table 1**).

**Table 1:** Characteristics of 28 patients with metastatic hepatic carcinoma treated by CSM-TACE who had regular Th1/Th2 and Th17 tests.

Clinical features	Data
Age (average age)	59.9 ± 9.26
Gender	
Male	16
Female	12
Liver metastasis type	
Simultaneity	7
Anisochronism	21
Number of liver metastases	
<3	4
3-5	15
>5	9
Tumor size	
3-5cm	10
>5cm	18
Extrahepatic metastasis	
Yes	15
No	13
Child classification of liver function	
A	24
B	4
PS score	
0	20
1	7
2	1
Primary tumor location	
Gastrointestinal cancer	20
Gynecologic Oncology	4
Lung cancer	2
Breast cancer	2

### Standardized CSM-TACE technology

After the successful puncture of the right femoral artery by Seldinger’s method, RH catheter was routinely introduced to perform abdominal hepatic artery and common hepatic artery angiography. The tumor supplying arteries were selected according to the angiographic findings and then compared with the preoperative imaging data to evaluate whether all intrahepatic lesions and blood vessels were found. The 1.0 g CalliSpheres drug-loaded microspheres with a diameter of 300-500 μm were used to load epirubicin 60mg, and the microspheres were mixed with chemotherapy drugs at room temperature, shake once every 5 minutes, and loaded for 30 minutes. Then mixed with non-ionic contrast agent iodixanol injection in 1:1 ratio, added gentamicin 80,000 units after mixing. The above mixture was diluted 10-20 times with water for injection and then slowly injected into the tumor supply artery. Uniform criteria for the endpoint of interventional embolization: angiography showed the tumor staining disappeared, the tumor target vessel was truncated, or the contrast agent remained in 3 cardiac cycles.

### The detection method of peripheral blood cytokines and CD4+T/CD8+T

Serum was collected for Th1 (interleukin 2 (IL-2), tumor necrosis factor-α (TNF-a), interferon (IFN-r)), Th2 (IL-4, IL-6, IL-10), Th17 (IL-17A), and other cytokines detection before CSM-TACE, and 2d and 5d after CSM-TACE. The venous blood samples were collected in standard test tubes or test tubes with separation gel, coagulated naturally at room temperature, or centrifuged at 2000-4000rpm for 20 minutes, and about 0.5ml of separated serum was taken for examination. Samples to be tested should be processed and tested within 4 hours of blood draw. Fluorescence detection was performed on the calibrated flow cytometer according to the sequence of standard control tube, negative control tube, and sample control tube. Each test tube was required to be tested immediately after vortex mixing for 3-5 seconds. 5mL of peripheral blood was collected with heparin sodium anticoagulant before CSM-TACE, 2d, and 5d after CSM-TACE, and the level of CD4^+^T/CD8^+^T was determined by the flow fluorescence method.

### Statistical analysis

The statistical analysis was conducted by SPSS software (provided by IBM, version 20.0). Results were expressed as mean ± SD. The t-test was used to compare the data of different groups. Non-normal data are represented in media and using rank-sum tests. The test level was a=0.05, and *P* < 0.05 was considered statistically significant.

## Results

### The Th1/Th2 level in peripheral blood of MHC before and after CSM-TACE

**Figure 1:**
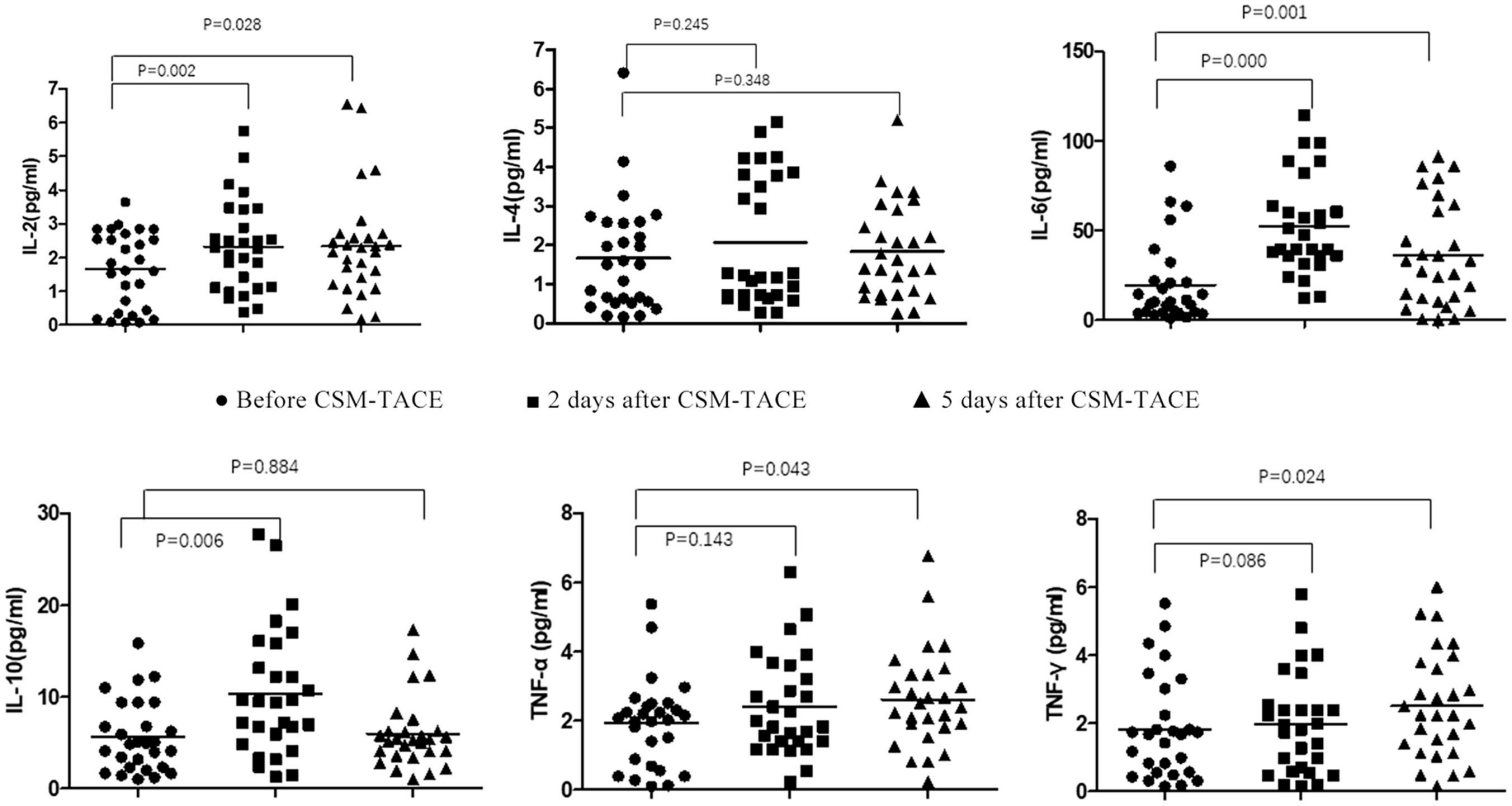
Flow Cytometry Chart: Changes of Th1/Th2 and IL-17A and CD4^+^/CD8^+^T in peripheral blood of metastatic hepatic carcinoma patients before and after CSM-TACE.

Before CSM-TACE, IL-2 level was 1.73(0.08-3.65)pg/ml, IL-4 level was 1.50(0.16-6.41)pg/ml, AND IL-6 level was 10.46(1.28-86.11)pg/ml. The levels of IL-10, TNF-A and IFN-R were 4.93(1.03-15.90)pg/mL, 2.05(0.10-5.38)pg/ml, and 1.68(0.16-5.53) pg/ml. The level of IL-6 increased 2d and 5d after CSM-TACE, which was statistically different from that before CSM-TACE (P < 0.05). The IL-10 level at 2 days after CSM-TACE was higher than that before CSM-TACE (P < 0.05), and decreased at 5 days after CSM-TACE, and was lower than that before CSM-TACE, and the difference was not statistically significant (P > 0.05). The IL-4 level showed a transient decline and then increased again, but there was no statistical difference (P > 0.05). The level of TNF-a decreased temporarily and then increased again and was higher than the preoperative level. There was a statistically significant difference in TNF-a before CSM-TACE and 5 days after CSM-TACE (P < 0.05). The postoperative IFN-r level showed a continuous and slowly increasing trend, and the IFN-r level on the 5th day after the CSM-TACE was significantly different from that before CSM-TACE (P < 0.05). The IL-2 level showed a trend of transient increase and then decreased again. Nevertheless, IL-2 at 2 and 5 days after CSM-TACE were significantly different from those before CSM-TACE (P < 0.05) (**Table 2**, **Figure 1**).

**Table 2:** Comparison of Th1/Th2 and IL-17A in metastatic hepatic carcinoma patients before and after CSM-TACE.

Group	Time	Proportions [Median (Interval)]	P
Il-2(pg/ml)	Before CSM-TACE	1.73(0.08-3.65)	
	2 days after CSM-TACE	2.27(0.38-5.75)	0.002
	5 days after CSM-TACE	2.24(0.17-6.54)	0.028
Il-4(pg/ml)	Before CSM-TACE	1.50(0.16-6.41)	
	2 days after CSM-TACE	1.21(0.26-5.15)	0.245
	5 days after CSM-TACE	1.52(0.25-5.20)	0.348
Il-6(pg/ml)	Before CSM-TACE	10.46(1.28-86.11)	
	2 days after CSM-TACE	43.69(12.55-114.40)	0.000
	5 days after CSM-TACE	30.38(0.495-91.39)	0.001
Il-10(pg/ml)	Before CSM-TACE	4.93(1.03-15.90)	
	2 days after CSM-TACE	9.47(1.30-27.73)	0.006
	5 days after CSM-TACE	5.33(1.09-17.29)	0.884
TNF-a(pg/ml)	Before CSM-TACE	2.05(0.10-5.38)	
	2 days after CSM-TACE	1.90(0.24-6.30)	0.143
	5 days after CSM-TACE	2.45(0.24-6.78)	0.043
IFN-r(pg/ml)	Before CSM-TACE	1.68(0.16-5.53)	
	2 days after CSM-TACE	1.89(0.16-5.79)	0.086
	5 days after CSM-TACE	2.24(0.16-6.01)	0.024
Il-17A(pg/ml)	Before CSM-TACE	21.64(5.06-32.82)	
	2 days after CSM-TACE	11.77(2.01-26.84)	0.000
	5 days after CSM-TACE	6.38(0.18-15.89)	0.000
CD4/CD8	Before CSM-TACE	1.48(0.39-4.11)	
	2 days after CSM-TACE	1.93(0.92-4.63)	0.0002
	5 days after CSM-TACE	2.63(1.61-5.33)	0.0005

### The Th17 level in peripheral blood of MHC before and after CSM-TACE

**Figure 2:**
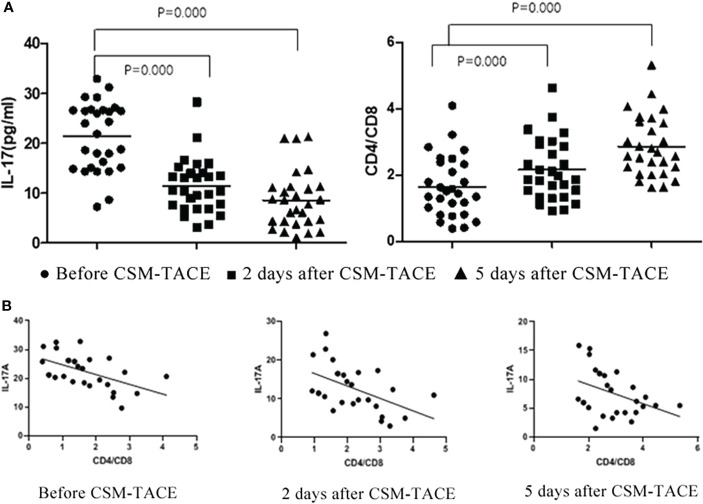
**(A)** Flow Cytometry Chart: Changes of IL-17A and CD4^+^/CD8^+^T in peripheral blood of metastatic hepatic carcinoma patients before and after CSM-TACE; **(B)** Correlation of IL-17A level and CD4^+^/CD8^+^T changes before and after CSM-TACE.

The level of IL-17A before CSM-TACE was 21.64(9.65-32.82)pg/ml, and the level of IL-17A began to decrease gradually at 2d and 5d after CSM-TACE, which were 11.77(3.01-26.84)pg/mL and 6.38(1.47-15.89)pg/ml, respectively, and the differences were statistically significant compared with those before CSM-TACE (P < 0.05) (**Table 2**, **Figures 2**).

### The CD4+/CD8+ proportion in peripheral blood of MHC before and after CSM-TACE

CD4^+^/CD8^+^T was 1.48(0.39-4.11) before CSM-TACE, and the CD4^+^/CD8^+^T gradually increased to 1.93(0.92-4.63) and 2.63(1.61-5.33), respectively at 2 and 5 days after CSM-TACE, and the levels at 2 and 5 days after CSM-TACE was still higher than those before CSM-TACE, and the differences were statistically significant (P < 0.05) (**Table 2**, **Figure 2**).

### Correlation of IL-7A level and CD4^+^/CD8^+^ proportion changes

After CSM-TACE, CD4^+^/CD8^+^T showed a gradually increasing trend, and IL-17A showed a gradually decreasing trend. According to statistics: there was negative correlation between CD4^+^/CD8^+^T and IL-17A (preoperative r = 0.536 p = 0.003, 2d after CSM-TACE r = 0.507 p = 0.006, 5d after CSM-TACE r = 0.395 p = 0.037) (**Figure 2**).

### Imaging changes of MHC before and after CSM-TACE

**Figure 3:**
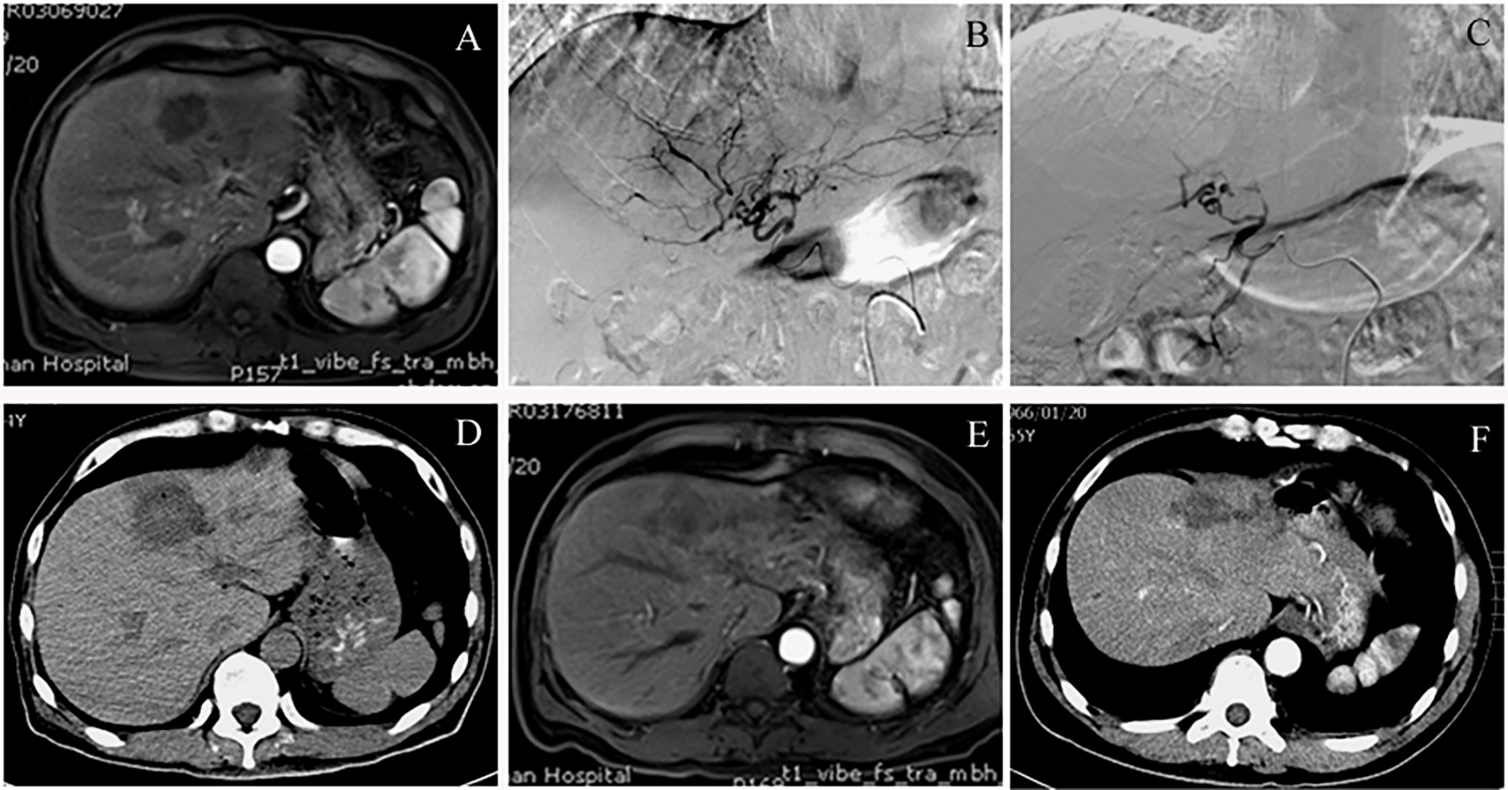
** **A case of colon cancer with liver metastases, convalescent period of cerebral hemorrhage and not suitable for chemotherapy. **(A)** Preoperative contrast-enhanced MRI of the upper abdomen showed multiple intrahepatic space-occupying lesions. The picture shows the lesions in segment 4 of the left lobe of the liver. In the arterial phase, the tumor showed borderline flaky enhancement. **(B)** Intraoperative angiography of CSM-TACE revealed a hepatic tumor that was clumpy and slightly stained, and was supplied by a branch of the left hepatic artery. **(C)** Angiography after embolization showed that the tumor staining disappeared and the branches of the hepatic artery were truncated. **(D)** Review CT scan at 4 days after CSM-TACE showed that the liver tumor was significantly low-density and the outline of the lesion was clearly visible. **(E)** Contrast-enhanced MRI at 3 months after CSM-TACE showed no obvious enhancement of the left hepatic tumor. **(F)** Contrast-enhanced CT at 6 months after CSM-TACE showed significant shrinkage of the tumor in the left lobe of the liver without enhancement in the arterial phase.

Preoperative enhanced MRI or CT showed enhanced tumor margins or weakly enhanced tumor parenchyma. Intraoperative DSA showed that the tumor was in mild to moderately advanced arterial staining, while the angiography after CSM-TACE showed tumor staining disappeared and the blood supply artery was cut off. A plain CT scan at 4 days after CSM-TACE showed clear low-density changes in liver tumors, and honeycomb necrosis was observed in the tumors in some cases. The ORR at 1 month after CSM-TACE reached 100%. **Figure 3** is the image for a representative MHC patient.

## Discussion

The results of this study showed that IL-6 increased significantly at 2 days after CSM-TACE and began to decrease at 5 days after CSM-TACE, which was consistent with the peak of IL-6 at 3days after TACE for liver cancer reported by Jekarl et al. (7). In this report, they also believed that among the tested cytokines, IL-6 and IL-22 appear to play a regenerative role. IL-6 is a pluripotent cytokine that can regulate various cellular functions, including cell proliferation, cell differentiation, immune defense mechanism, and hematopoiesis, etc., and is closely related to the occurrence and development of various tumors, thus affecting the progression of tumors (5, 6). In this study, CD4^+^/CD8^+^T increased gradually after CSM-TACE, and TH1(IL-2, IFN-R, TNF-A) levels on the 5th day after CSM-TACE were significantly higher than the preoperative levels, which further demonstrated that the immune microenvironment of the body showed a trend of enhancing specific anti-tumor ability after CSM-TACE. The duality of immune inflammation will also become a golden period for the body to promote tumor proliferation after CSM-TACE, because it will increase the levels of a series of inflammatory factors that promote tumor growth, including SCF, M-CSF, G-CSF, GM-CSF, VEGF, COX-2 and PGE2, etc. (18), these repair factors will up-regulate the level of MDSCs (19), enhance the CTLA-4 pathway-dependent Treg/DC cell contact inhibition ability, reduce the body’s anti-tumor ability, and then cause tumor escape (20).

IL-17A with double-sided characteristics showed a gradual decline process after CSM-TACE in patients with liver metastatic carcinoma, and it was negatively correlated with CD4^+^/CD8^+^T, the main reason was that with the significant necrosis of liver tumors, the tumor activity gradually decreased, and the body’s anti-tumor ability increased, then the IL-17A level decreased. It has also been reported that IL-17 after TACE in PHC patients was increased, and it was positively correlated with the prognosis. The study results of Liao et al. showed an increased frequency of circulating Th17 cells at 30 days after TACE (Th17 D30) compared with the baseline level, and Th17 D30 was positively associated with overall survival, and Th17 is a potential prognostic marker for stage III HCC patients who underwent TACE (21). The changes in the body’s inflammatory factors caused by TACE are complex. This is because the immune network itself is complex. In particular, the different types of embolic agents used in TACE can lead to different degrees of tumor necrosis, which may also cause different types of immune-inflammatory reactions.

This study also showed that the IL-2 and IFN-r levels continued to increase after CSM-TACE, and the TNF-a level was also significantly higher than the preoperative level on the 5^th^ day after CSM-TACE. Therefore, although the recent changes in the immune-inflammatory network after CSM-TACE were complex, there was still overall consistency of changes, which further suggested that the simultaneous presence of acute inflammatory response and immune anti-tumor response may be a typical short-term postoperative CSM-TACE immunological phenomenon, the changes in IL-6 and Il-17A levels were more significant, but the changes in IFN-r and CD4^+^/CD8^+^T levels seem to be more specific. In addition, the chemotherapy drugs and dosages used in TACE may also cause different immune responses (22, 23). The chemotherapeutic drugs of CSM-TACE dominated by drug-loaded microspheres are slowly released in the liver tumor, which may be the main reason for its great difference from other conventional TACE.

There have been many studies on the correlation between changes in inflammatory cytokines and prognosis (24). This research mainly focused on the inflammatory cytokines in MHC patients within 1 week after CSM-TACE and observed the recent changes of tumor inflammatory microenvironment, and we found subtle changes within 1 week after CSM-TACE. The coexistence of acute inflammation and immune anti-tumor is a typical tumor microenvironment feature within 1 week after CSM-TACE. Meanwhile, the characteristics of immune inflammation caused by CSM-TACE provide us with a series of immunotherapy targets for reference, and 1 week after liver metastatic carcinoma surgery is also the best time point for comprehensive treatment after interventional surgery. These findings provide a theoretical basis for comprehensive treatment of liver metastatic carcinoma after CSM-TACE.

Due to the complexity of the body’s immune network and the mechanism of malignant tumors, the immune-inflammatory changes caused by TACE are also extremely complex and are fundamentally different from the immune changes caused by systemic therapy such as PD-1 inhibitors (25). Nevertheless, the results of this study showed that CSM-TACE not only had a good tumor local control rate but also resulted in the immunological phenomena of anti-tumor immune enhancement and acute inflammation coexist, which provided important immunological clues for the comprehensive treatment of liver metastases after CSM-TACE. Of course, further studies are needed to further confirm the effect of this method on the immune microenvironment and the mechanism. The limitations of this study are the small sample size and short research time limit. Therefore, a prospective large-sample multi-center study is needed to further explore the effects of CSM-TACE on immune inflammation and make contributions to basic and clinical translational research in the future.

## Data availability statement

The original contributions presented in the study are included in the article/supplementary material. Further inquiries can be directed to the corresponding authors.

## Ethics statement

The study was approved by the ethics committee of Affiliated Zhongshan Hospital of Dalian University. The patients/participants provided their written informed consent to participate in this study.

## Author contributions

YL and SL, responsible for clinical trial research and paper writing. XL and FG, responsible for patient follow-up and data statistics. ZR, JB, and JW, responsible for CD4^+^/CD8^+^ detection and CSM-TACE. YZ and GZ, responsible for project design and experimental implementation. All authors contributed to the article and approved the submitted version.

## Conflict of interest

The authors declare that the research was conducted in the absence of any commercial or financial relationships that could be construed as a potential conflict of interest.

## Publisher’s note

All claims expressed in this article are solely those of the authors and do not necessarily represent those of their affiliated organizations, or those of the publisher, the editors and the reviewers. Any product that may be evaluated in this article, or claim that may be made by its manufacturer, is not guaranteed or endorsed by the publisher.
